# Detection of *Leishmania infantum* DNA in *Pintomyia evansi* and *Lutzomyia longipalpis* in Honduras

**DOI:** 10.1186/s13071-020-04462-y

**Published:** 2020-11-23

**Authors:** Wilfredo Sosa-Ochoa, Javier Varela Amador, Yokomi Lozano-Sardaneta, Gabriela Rodriguez Segura, Concepcion Zúniga Valeriano, Gabriela Venicia Araujo, Carmen María Sandoval Pacheco, Márcia Dalastra Laurenti, Fredy Galvis-Ovallos

**Affiliations:** 1 Microbiology Research Institute, Universidad Nacional Autónoma de Honduras, Tegucigalpa, Honduras; 2 Hospital Militar de Honduras, Tegucigalpa, Honduras; 3 Centro de Medicina Tropical, División de Investigación, Facultad de Medicina, Universidad Nacional Autónoma de México, Ciudad de México, México; 4 Health Surveillance Department, University School Hospital, Tegucigalpa, Honduras; 5 Laboratory of Pathology of Infectious Diseases, Medical School, São Paulo University, São Paulo, SP Brazil; 6 Department of Epidemiology, School of Public Health, University of São Paulo, São Paulo, Brazil

**Keywords:** *Leishmania* (*Leishmania*) *infantum*, *Pintomyia* (*Pifanomyia*)* evansi*, *Lutzomyia* (*Lutzomyia*) *longipalpis*, Visceral leishmaniasis, Non-ulcerative cutaneous leishmaniasis

## Abstract

**Background:**

The two most abundant sand fly species on the Honduran Pacific coast are *Lutzomyia* (*Lutzomyia*) *longipalpis* and *Pintomyia* (*Pifanomyia*) *evansi*. Both species are known vectors of *Leishmania* (*Leishmania*) *infantum*, the etiological agent of visceral leishmaniasis (VL) in the Americas. Although VL and non-ulcerative cutaneous leishmaniasis (NUCL) are endemic on the Pacific versant of the Central American Pacific, the latter is the most frequent manifestation of leishmaniasis there. We evaluated the circulation of *Leishmania* spp. in the sand fly species on El Tigre Island, an endemic area of NUCL.

**Results:**

We collected 222 specimens of six sand fly species. *Lu. longipalpis* (180 specimens; 81%) and *Pif.* (*Pi*.) *evansi* (35 specimens; 16%) were the most abundant species. *L*. (*L*.)* infantum* DNA was detected in nine of the 96 specimens analyzed; seven of these specimens were identified as *Lu. longipalpis,* and the remaining two were *Pi.* *evansi*, with an infection rate of 9.4% and 2.7%, respectively.

**Conclusion:**

We present the first record of *L.* (*L.*) *infantum* DNA in *Pi.* *evansi* from a NUCL endemic region of Central America. Our results suggest that *Pi. evansi* could be a secondary vector of* L*. (*L*.)* infantum* in the transmission cycle of leishmaniasis*.* The detection of natural infections of *L.* (*L*.) *infantum* in sand flies in this region contributes to an understanding of the epidemiology of leishmaniasis in Honduras.
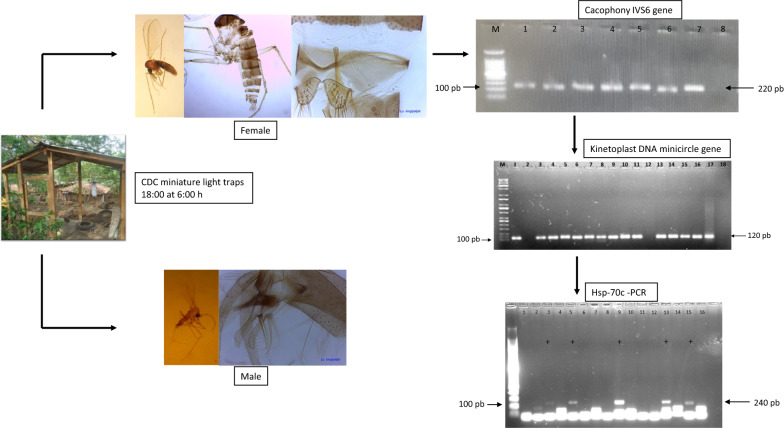

## Background

Leishmaniasis is a vector-borne parasitic disease caused by species of the genus *Leishmania* (Kinetoplastida: Trypanosomatidae). *Leishmania* is widespread mainly in tropical and subtropical regions of 98 countries throughout Europe, Africa, Asia, and America [1]. More than 1,000 sand fly species have been identified worldwide, of which 530 species are present in the Americas [2, 3], and at least 30 species are considered *Leishmania* vectors [4]. In Honduras, 29 sand fly species have been reported [2, 5–7]. Members of the *Lutzomyia longipalpis* species complex are the main vectors of *Leishmania* (*Leishmania*) *infantum*; however, other sand fly species, including *Lutzomyia* (*Lutzomyia*) *cruzi* and *Pintomyia* (*Pifanomyia*) *evansi*, play a role as vectors of this parasite in some endemic areas of visceral leishmaniasis (VL) in South and Central America [8, 9]. *Lu. longipalpis* s.l. is the most abundant sand fly species in the area endemic for VL and non-ulcerative cutaneous leishmaniasis (NUCL) in the southern region of Honduras [6, 10], and is the only species reported as a vector of *L.* (*L*.) *infantum* [11]. Nevertheless, the role of *Pi. evansi* in VL transmission dynamics in Honduras is unknown, although evidence of its vector competence has been reported in South America [12]. For this reason, the aim of this study was to evaluate the DNA circulation of *L*. (*L*.)* infantum* in *Lu. longipalpis* and *Pi.* (*Pif.*) *evansi* on a Mesoamerican Pacific island.

## Methods

### Study area and sand fly collection

This study was carried out in Amapala municipality (13°17′26.082″N, 87°39′5.543″W), Valle department, an area covering 80.7 km^2^. The municipality comprises two islands, Zacate Grande and El Tigre, located in the Gulf of Fonseca in southern Honduras. Sand flies were sampled for five consecutive nights in May 2018 from five localities: Las Pelonas (13°16′58.332″N, 87°37′4.799″W), Punta Honda (13°16′21.576″N, 87°36′56.879″W), Tigüilotada (13°15′45.720″N, 87°36′57.527″W), Islitas (13°15′39.456″N, 87°37′24.276″W) and Playa Grande (13°16′4.183″N, 87°39′31.104″W). The captures were carried out from 6:00 p.m. until 6:00 a.m., using automatic CDC miniature light traps (model 512; John W. Hock, Gainesville, FL) in neighborhoods where active cases of NUCL were evident. The traps were installed in peridomiciliary environments, near decomposing organic matter or next to latrines. The specimens were separated and processed 1 day after capture.

### Taxonomic identification of the sand fly species

The taxonomy of the phlebotomine sand flies was conducted by analyzing their morphological characteristics. In adherence to the identification procedures outlined by Mejía et al. [6], the sand flies were first mounted and the specific species identified in accordance with Young and Duncan [5]. Finally, Galati et al. [13] were the primary academic resource used during the classification stage of the genera and the species.

### Genomic material extraction and polymerase chain reaction

Genomic DNA was extracted from gut tissues dissected from individual female sand flies using the Chelex 100 Kit (Bio-Rad, Hercules, CA). As an internal control for the DNA extraction, the cacophony* IVS6* gene present in the sand fly genome was amplified [14]. For the detection of the genus *Leishmania*, we used the primers Leish1: 5ʹ-AACTTTTCTCTGGTCCTCCGGGTAG-3ʹ and Leish2: 5′-ACCCCCAGTTTCCCGCC-3ʹ to amplify an approximate 120-base pair (bp) product [15]. Amplifications were performed using a commercial kit (Master Mix 2X; Promega). Each reaction was performed by adding 4 µl of target DNA and 0.6 µmol/L of each primer to obtain a final volume of 20 µl. The polymerase chain reaction (PCR) reactions were done in an Applied Biosystem 2770 Thermal Cycler (ThermoFisher Scientific, USA), under the following conditions: initial denaturation cycle at 94 °C for 5 min, followed by 35 cycles at 94 °C for 15 s, 60 °C for 20 s and 72 °C for 60 s, and final extension at 72 °C for 10 min. The amplification products were analyzed by electrophoresis in 1.5% agarose gel.

To characterize the *Leishmania* species, PCR-restriction fragment length polymorphism (RFLP) was performed, which amplified a specific region of the* hsp70* gene [16]. The primers used were* hsp70* sense (5' GGACGAGATCGAGCGCATGGT 3') and* hsp70* antisense (5' TCCTTCGACGCCTCCTGGTTG 3'). The reaction mixture was prepared in a final volume of 20 µl with 10 µl Master Mix 2X (Promega), 4 µl of target DNA and 0.6 µmol/L of each primer. The PCR reactions were done under the following conditions: initial denaturation at 94 °C for 5 min, followed by 37 cycles at 94 °C for 30 s, 61 °C for 1 min and 72 °C for 3 min, and a final extension cycle at 72 °C for 10 min. The amplification products were analyzed by electrophoresis in 2% agarose gel. To perform the restriction of the PCR products, the restriction enzyme HaeIII (Promega) was used, 5 µl of amplified DNA was added to the reaction and the mixture incubated at 37 °C for 3 h. The species profiles of each sample and reference controls were observed in 4% of agarose gel subjected to electrophoresis for 3.5 h.

## Results and discussion

A total of 222 sand fly specimens were collected, which were predominately males (66%) (Table 1). Six species were identified by using morphological characters. The most predominant sand fly species collected was *Lu.* (*Lu.*) *longipalpis*, followed by *Pi* (*Pif.*) *evansi*. The other species that were captured included *Micropygomyia* (*Micropygomyia*) *cayennensis cayennensis*, *Micropygomyia* (*Coquillettimyia*) *chiapanensis*, *Dampfomyia* (*Coromyia*) *beltrani* and *Lutzomyia* (*Tricholateralis*)* gomezi*. *Lu.* (*Lu.*) *longipalpis* has been previously studied in the region and was incriminated as the vector of *L*. (*L*.)* infantum* [11]. The behavioral characteristics of this species in the study area were described by Carrasco et al. [7]. Recently, Mejía et al. [6] expounded on various aspects of sand flies’ feeding preferences within the Pacific Honduran area. These authors observed a predominance of *Pi.* (*Pif.*) *evansi* and *Lu.* (*Lu.*) *longipalpis*, but did not detect the presence of *L*. (*L*.)* infantum* in these species [6]. These sand flies were also predominant in other NUCL-endemic areas of Central America (i.e., Costa Rica and Nicaragua) [17, 18]

**Table 1:** Sand fly species captured, by locality, and detection of *Leishmania* spp. and *Leishmania* (*Leishmania*)* infantum*

Locality (sand flies; *n*)	Species	Males (*n*)	Females (*n*)	%	Specimens with *Leishmania* spp. DNA (*n*) (%)	Females with *L.* (*L.*)* infantum* DNA (*n*) (%)
Playa Grande (175)	*Lutzomyia* (*Lutzomyia*) *longipalpis*	95	35	79.2	26 (35.13)	7 (9.45)
	*Pintomyia* (*Pifanomyia*)* evansi*	10	24	20.8	11 (14.8)	2 (2.70)
Las Pelonas (16)	*Lutzomyia* (*Lutzomyia*) *longipalpis*	12	2	87.5	–	–
	*Micropygomyia* (*Micropygomyia*)* cayennensis cayennensis*	1	1	12.5	–	–
Punta Honda (29)	*Lutzomyia* (*Lutzomyia*) *longipalpis*	24	4	96.5	–	–
	*Micropygomyia* (*Coquillettimyia*)* chiapanensis*	0	1	3.5	–	–
Islitas (13)	*Lutzomyia* (*Lutzomyia*) *longipalpis*	6	2	61.5	–	–
	*Dampfomyia* (*Coromyia*)* beltrani*	0	2	15.4	–	–
	*Pintomyia* (*Pifanomyia*)* evansi*	0	1	7.7	–	–
	*Micropygomyia* (*Micropygomyia*)* cayennensis cayennensis*	0	1	7.7	–	–
	*Lutzomyia* (*Tricholateralis*)* gomezi*	0	1	7.7	–	–

**Fig. 1:**
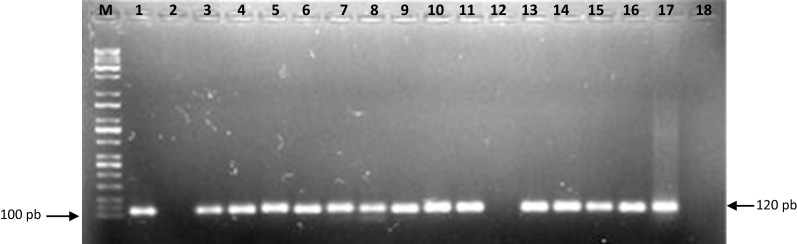
Polymerase chain reaction (PCR) to determine *Leishmania* spp. infection using Leish1-Leish2 primers to target conserved DNA regions of the kinetoplast DNA from *Leishmania* spp. [120 base pairs (bp)].* Lane M* Molecular weight marker (100-bp DNA ladder).* Lanes 1–16* Female sand fly DNA [*lanes 1-10* *Lutzomyia* (*Lutzomyia*) *longipalpis*, positive female;* lanes 11*,* 13–16* *Pintomyia* (*Pifanomyia*)* evansi*, positive female].* Lane 17* PCR positive control [DNA extracted from a mixture of the male insect pool containing *Leishmania* (*Leishmania*)* infantum* DNA].* Lane 18* Amplification reaction without added DNA (PCR negative control)

Thirty-seven of the 96 analyzed female specimens were positive for the genus *Leishmania* according to the PCR results (Fig. 1). *L.* (*L.*) *infantum* DNA was revealed in nine specimens: seven *Lu.* (*Lu.*) *longipalpis* and two *Pi.* (*Pif.*) *evansi*. The *L*. (*L*.)* infantum* infection rate was 9.4% for *Lu.* (*Lu.*) *longipalpis* and 2.7% for *Pi.* (*Pif.*) *evansi*. All of the samples analyzed produced an amplified product of 220 bp, corresponding to a *Lutzomyia* spp. constitutive gene (cacophony), which confirmed the integrity of the insect DNA preparation and the absence of PCR inhibitors [14]. We used a method for the detection of *Leishmania* spp. described in Francino et al. [15]. However, one limitation of this method is the small size of the PCR product (120 bp), which makes sequencing unlikely. Therefore, the method described by Graça et al. [16] was used to differentiate *Leishmania* species. This method amplifies a *Leishmania* 234-bp* hsp70* fragment and shows similar sensitivity to the PCR-internal transcribed spacer 1 (>70%) method used to detect *Leishmania* DNA; however, associating this 234-bp* hps70* with a RFLP protocol may give researchers the advantage of being able to distinguish this *Leishmania* species by electrophoresis [19].

Our study is the first to report the presence of *L*. (*L*.)* infantum* DNA in *Pi. evansi* females in Central America. In two studies in Colombia, in endemic areas of VL, the natural infection rate of *L*. (*L*.)* infantum* in *Pi. evansi* was found to be 0.10% [12] and 0.34% [19]. The natural infection rates of *Lu. longipalpis* were between 0.5% and 1.1% according to direct observations of dissected intestines [11, 20] and from PCR of dissected intestines [21–23]. We report a 9.4% infection rate of* L*. (*L*.)* infantum* in *Lu. longipalpis*, which is in agreement with other research [22, 23]. Although the detection of *Leishmania* DNA in sand flies does not indicate the ability of these species to transmit this parasite, we evidenced contact between *Pi. evansi* and *Lu. longipalpis* with the natural host of *L*. (*L*.)* infantum* in the study area, in the Amapala municipality. Considering that the vector competence of *Pi. evansi* has been previously described [12], it is probable that both *Lu. longipalpis* and *Pi. evansi* transmit *L*. (*L*.)* infantum* in southern Honduras.

## Conclusion

We present for the first time evidence of the presence of *L*. (*L*.)* infantum* DNA in *Pi. evansi* in a NUCL endemic region of Central America. Considering the natural infection of *Lu. longipalpis* by *L*. (*L*.)* infantum*, our results indicate that *Pi. evansi* might be a secondary vector of this parasite, and might be involved in the transmission cycle of leishmaniasis. The detection of natural infections of *L*. (*L*.)* infantum* in sand flies in this region contributes to our understanding of the epidemiology of leishmaniasis in Honduras.

## Data Availability

Data supporting the conclusions of this article are included within the article. Specimens of *Lutzomyia* analyzed during the study are available from the corresponding author upon reasonable request.

## Abbreviations

VL: Visceral leishmaniasis
NUCL: Non-ulcerative cutaneous leishmaniasis
PCR: Polymerase chain reaction
bp: Base pairs
RFLP: Restriction fragment length polymorphism
